# Effect of Thickness of Molybdenum Nano-Interlayer on Cohesion between Molybdenum/Titanium Multilayer Film and Silicon Substrate

**DOI:** 10.3390/nano9040616

**Published:** 2019-04-16

**Authors:** Huahai Shen, Bing Yao, Jianwei Zhang, Xinqiao Zhu, Xia Xiang, Xiaosong Zhou, Xiaotao Zu

**Affiliations:** 1 Institute of Nuclear Physics and Chemistry, China Academy of Engineering Physics, Mianyang 621900, China; yaob1974@sina.com (B.Y.); zhuxinqiao@zju.edu.cn (X.Z.); 2 School of Physics, University of Electronic Science and Technology of China, Chengdu 610054, China; jianweizhang19@163.com (J.Z.); xiaxiang@uestc.edu.cn (X.X.); xtzu@uestc.edu.cn (X.Z.)

**Keywords:** titanium film, interlayer, cohesion, residual stress, nano-indenter

## Abstract

Titanium (Ti) film has been used as a hydrogen storage material. The effect of the thickness of a molybdenum (Mo) nano-interlayer on the cohesive strength between a Mo/Ti multilayer film and a single crystal silicon (Si) substrate was investigated by X-ray diffraction (XRD), scanning electron microscopy (SEM), and nano-indenter. Four groups of Si/Mo/Ti multilayer films with different thicknesses of Mo and Ti films were fabricated. The XRD results showed that the introduction of the Mo layer suppressed the chemical reaction between the Ti film and Si substrate. The nano-indenter scratch results demonstrated that the cohesion between the Mo/Ti film and Si substrate decreased significantly with increasing Mo interlayer thickness. The XRD stress analysis indicated that the residual stress in the Si/Mo/Ti film was in-plane tensile stress which might be due to the lattice expansion at a high film growth temperature of 700 °C and the discrepancy of the thermal expansion coefficient between the Ti film and Si substrate. The tensile stress in the Si/Mo/Ti film decreased with increasing Mo interlayer thickness. During the cooling of the Si substrate, a greater decrease in tensile stress occurred for the thicker Mo interlayer sample, which became the driving force for reducing the cohesion between the Mo/Ti film and Si substrate. The results confirmed that the design of the Mo interlayer played an important role in the quality of the Ti film grown on Si substrate.

## 1. Introduction

Thin film materials maintain the fantastic properties of bulk materials [1,2,3,4,5,6] and have the significant advantages of being more economic [7], having small device size [4,5] and having the new physical performance of two-dimensional material [8,9,10]. Thin film materials have been used as functional devices in the applications of semi-conductors [2,8,9,10], metal hydrides [1,4,5,11,12,13], and solar cells [14,15], etc. It is more conducive to the combination of multiple functional materials to form multilayer film materials [16,17], which can not only realize the unique characteristics of each material, but also give play to the characteristics of new interface materials [18,19,20] and improve their comprehensive performance [19,20,21,22,23]. Therefore, the design and fabrication of new multilayer functional materials have attracted attention as hot issues in current research [23]. Postolnyi et al. [23] has proposed that a lower individual layer thickness with smaller grain size and more interlayer interfaces can significantly improve the mechanical properties of CrN/MoN multilayers. Li et al. [18] has found that smaller layer thickness is beneficial in reducing the irradiation hardening of Fe/W multilayers. Pshyk et al. [24] synthesized multilayer TiAlSiYN/MoN coatings that had improved mechanical properties in comparison to a single-layer TiAlSiYN nanocomposite film. The cohesive strength between multilayer and substrate is another crucial problem in multilayer film design and is affected by substrate roughness [25], substrate temperature [25,26], different thermal expansion coefficient in the film-substrate couple [27,28], and film thickness [29,30,31], etc. A novel nanoindentation technique has been proposed and was employed to test the nanomechanical properties of multilayered Al_2_O_3_/TiO_2_ nanolaminates [32]. A theoretical method based on density functional calculation (DFT) has been developed to study film structure stability and the cohesion between the thin film and substrate [33]. Goyenola et al. [33] has addressed stable compounds of fullerene-like sulpho-carbide and obtained their geometry optimizations and cohesive energies. Ren et al. [34] investigated the strain and cohesive energy of a TiN film on an Al(001) substrate using the DFT method and found that the TiN film could be steadily deposited on the AlN(001) interface. 

Titanium film has the advantages of good hydrogen absorption performance, high thermal stability, and low room temperature equilibrium pressure. Ti film has been used as a metal hydride in the solid phase and has played an important role in the fields of hydrogen storage [35], solar thermal energy storage [36], and nuclear energy storage [4,37,38,39]. It has been reported that the surface morphology and grain size of Ti film on a Mo substrate was severely affected by the heterogeneity of polycrystalline Mo [4,38,40]. The hydrogenation performances of Ti films have been strongly correlated to their grain size and density of grain boundary. Thus, the design of new substrates for high quality Ti film growth is in demand. Single crystal Si is a candidate substrate material due to its properties of high thermal and electrical conductivity [5,11,41]. However, the chemical reaction between Ti and Si to form a Ti-Si complex at high substrate temperature restricts its application [42,43]. Shen et al. [13] have reported the formation of an Er-Si complex in Er film grown on Si. It has been demonstrated by Parish et al. [5,11] that the reaction between the Er film and Si substrate was suppressed by the introduction of a Mo interlayer between the Er film and Si substrate. 

Screening and designing membrane material systems and film thicknesses are of great importance for the fabrication of high quality multilayer materials [18,23]. However, extensive study on the relationship between the cohesion of the film-substrate couple and film thickness has rarely been reported to date [29,30,31]. Based on the important application prospects of multilayer films in many fields [16,18,22,23], this work focuses on the influence of a Mo interlayer on the cohesion of an Si-based Ti film. The cohesion between the Ti film and the Si substrate has been discussed in relation to the residual stress [6,44,45] of the Ti film and the different thermal expansion coefficients of the Ti and Si materials [27,28].

## 2. Experimental Details

### 2.1. Film Growth and Sample Preparation

Ti films and Mo interlayers were electron beam evaporated onto polished single crystal Si (110) substrates by using source materials of Ti bulk (PrMat, Shanghai, China, 99.99% purity, 25 mm in diameter) and Mo bulk (PrMat, Shanghai, China, 99.99% purity, 25 mm in diameter) [4,13]. Before deposition, the Si substrates were ultrasonically cleaned in ethanol and acetone and outgassed at 700 °C for 2 h in a chamber with a base vacuum pressure better than 2 × 10^−4^ Pa. The sample holder was rotated during deposition to obtain uniform films and the distance between source materials and sample holder was ~20 cm. The Mo interlayer was first grown on the Si substrate at a temperature of 500 °C, followed by Ti film evaporation on the Mo layer to form Si/Mo/Ti multilayer films. The Ti films were grown at a temperature of 700 °C to avoid the severe oxidation of the Ti-based getter material [4,46]. The evaporation rates detected by the IC5 thin film deposition controller were 15 nm/s and 2 nm/s for the Ti and Mo films, respectively. Four kinds of Si/Mo/Ti films were fabricated with different thicknesses of Mo interlayers by controlling the deposition time from 1 min to 8 min. The deposition parameters and sample designation of the as-grown multilayer films are summarized in Table 1. The substrate temperature was naturally cooled to room temperature immediately after the Mo and Ti film evaporations. 

### 2.2. Sample Characterizations

The surface morphology of the multilayer films was acquired by SEM using a Zeiss Auriga workstation (Carl Zeiss Microscopy GmbH, Jena, Germany) equipped with X-ray energy dispersive spectroscopy (EDS) and a gallium focused ion beam (FIB). The elemental distributions of Ti, Mo, and Si elements on the film surface and along the cross-sectional structure were collected by EDS using an Oxford X-Max^N^ 150 mm^2^ (Oxford Instruments, Abingdon, UK). Cross-sectional SEM samples of the Si/Mo/Ti films were prepared to measure the thicknesses of the Mo and Ti films by using an advanced FIB method working in the SEM facility [47]. A large stair-step trench was milled out at the interested area from the film surface to expose the cross-sectional structures of the Si/Mo/Ti films. The crystal structures of the Si/Mo/Ti films were analyzed by XRD using an X’Pert PRO MPD (PANalytical B.V., Almelo, The Netherlands) with Cu K*α* irradiation working at 45 kV and 40 mA. The residual stress of the Si/Mo/Ti films was analyzed by a classical sin^2^*ψ* method using XRD equipment [12,23]. Ten *θ*-2*θ* scans were performed at a fixed *ψ* position by tilting the *ψ* angle from 0° (the sample normal direction) to 36°. The Ti (112) diffraction peak positioned at ~76.3° is preferred for residual stress analysis considering the peak intensity and accuracy of stress results. Therefore, each *θ*–2*θ* scan was collected from 74.5° to 79.5° using the typical parameters of 0.013° in step size and 80 s in count time. 

The cohesion between the Mo/Ti film and Si substrate was tested using an Agilent G200 Nano Indenter (Agilent, Santa Clara, United States) working with the scratch mode [48,49,50]. The scratch tests involved dragging a nano-indenter across the surface of the film with a continuously increasing normal load until surface buckling occurred at the critical load. The scratch experiment was explored by moving the indenter along the film surface for the first time to check the surface smoothness, followed by scratching the film with a linearly increasing normal force to a maximal load of 150 mN over a total scratch length of 500 μm [49]. The distance between the scratches was set to 100–600 μm and the scratch velocity was set to 50 μm/s. The SEM and EDS analyses were carried out on five scratch tracks for each sample to evaluate the cohesion between the film and substrate. 

## 3. Results and Discussions

**Figure 1:**
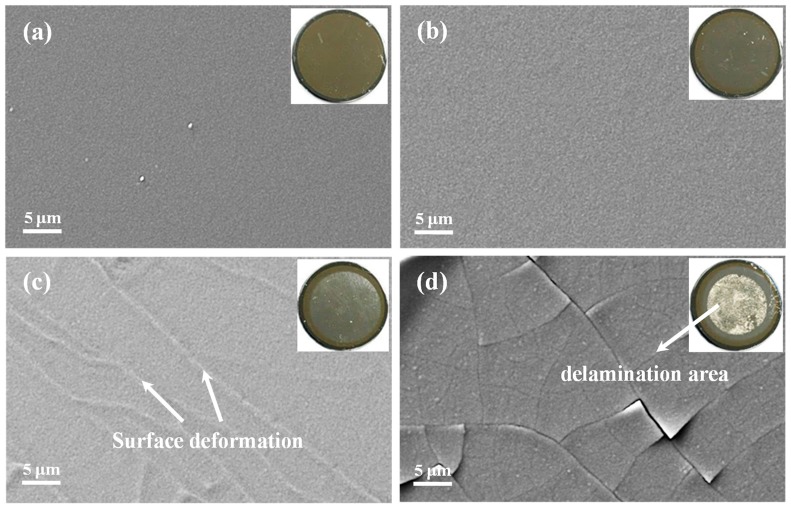
SEM and optical microscopic images of as-grown Si/Mo/Ti multilayer films with different Mo transition layer thicknesses for (**a**) Mo-1, (**b**) Mo-2, (**c**) Mo-4, and (**d**) Mo-8. The Mo-1 and Mo-2 samples exhibited smooth surfaces while the Mo-4 sample started to display surface deformation and the Mo-8 sample showed obvious surface delamination.

Based on the Mo interlayer and deposition times of 1 min, 2 min, 4 min, and 8 min, the as-grown Si/Mo/Ti multilayer films on single crystal Si (110) substrates were designated Mo-1, Mo-2, Mo-4, and Mo-8, respectively. Figure 1 shows the SEM images of the Mo-1, Mo-2, Mo-4, and Mo-8 samples, where the insets are the corresponding optical images. As shown in Figure 1a,b, it is clear that the Mo-1 and Mo-2 samples were of high quality, with very smooth surfaces and nanometer grain sizes. When the deposition time of the Mo interlayer was increased to 4 min and 8 min, the surface deformation started to appear for the Mo-4 sample and the obvious surface delamination occurred in the center area of the Mo-8 sample (labeled by arrow, shown in Figure 1c,d).

**Figure 2:**
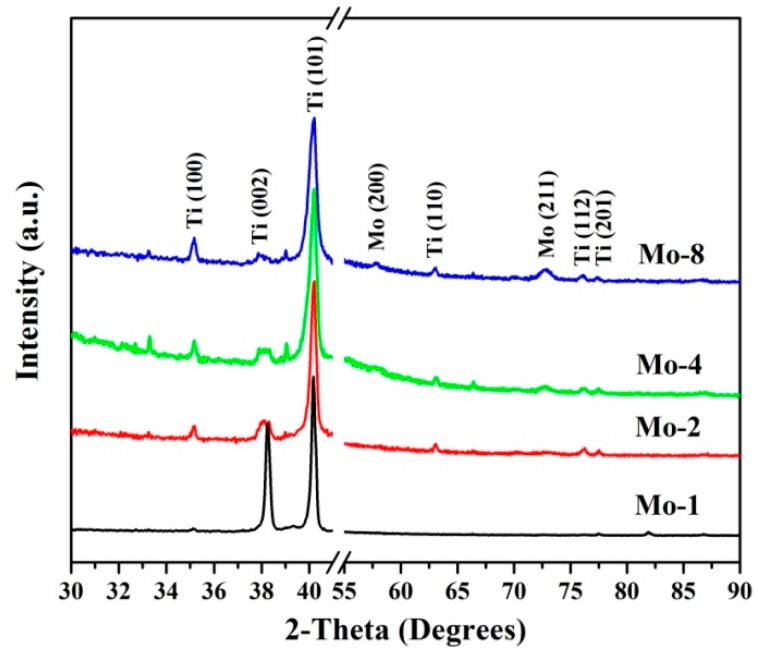
(Color online) X-ray diffraction (XRD) patterns of Si/Mo/Ti multilayer films with different Mo transition layer thicknesses. All four samples show strong Ti (101) preferred crystal orientation.

Figure 2 shows the XRD patterns of the Mo-1, Mo-2, Mo-4, and Mo-8 samples. The 2*θ* range from 42° to 55° is omitted to clearly present the diffraction peaks of the Ti and Mo crystals, since the Si (110) peak at 47.28° is much stronger than the other peaks. All the diffraction peaks could be well indexed as Ti and Mo have hexagonal-close packed (HCP) and body-centered cubic (BCC) crystal structures [4]. As shown in Figure 2, all four samples presented a strong preferred crystallographic orientation of Ti (101). Two tiny peaks positioned at 57.76° and 72.87° were indexed as Mo (200) and Mo (211) [4]. The relative intensities of the Mo peaks increased significantly with the Mo deposition time, revealing that the Mo layer thickness increased with deposition time. It is notable that no obvious peak of the Ti-Si compound [42,43] could be found in the XRD patterns of all four samples, demonstrating that the introduction of the Mo interlayer suppressed the chemical reaction between the Ti film and Si substrate, although the substrate temperature was higher than the onset temperature of 620 °C for the Ti-Si chemical reaction reported by Bensch et al. [11,43]. The Er-Si compounds started to form at a temperature above 350 °C [13] and could be well prevented by the introduction of an Mo interlayer with a thickness of about 100 nm [5]. The above results indicate that the pure Ti films were successfully grown on Si substrates, and the thickness of the Mo interlayer had a strong impact on the surface morphology and cohesion between the Mo/Ti film and Si substrate. 

**Figure 3:**
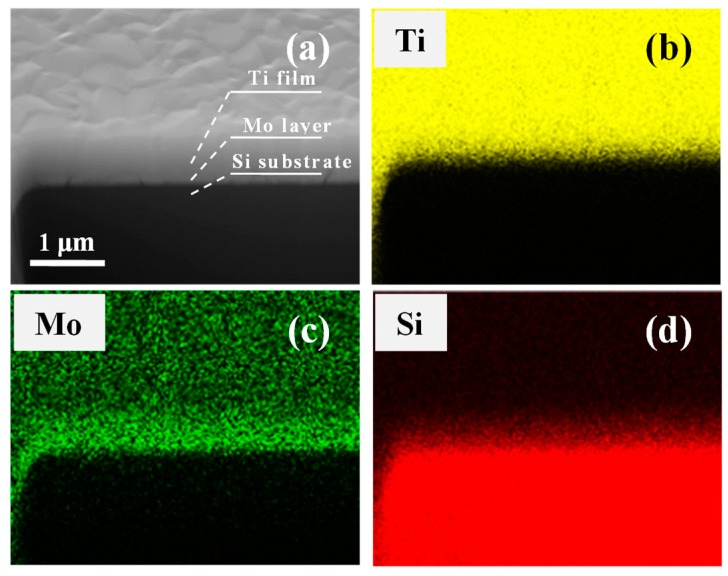
(Color online) (**a**) Cross-sectional SEM image of Mo-2 sample. (**b**–**d**) The Ti, Mo, and Si element distribution of (a) shows a clear Mo transition layer between the Ti film and Si substrate.

Figure 3a shows a representative cross-sectional SEM image of the Mo-2 sample, where the Si substrate, Mo interlayer, and Ti film could be identified and labeled. It is clear that the grain size of the Ti film is in nanometers and ranges from ~200 nm to ~400 nm. Figure 3b–d displays the EDS mapping results of the Ti, Mo, and Si element distributions seen in Figure 3a. As shown in Figure 3c, the signal of the Mo X-ray in the Mo interlayer is remarkably stronger than that in the Ti film, confirming the fabrication of the Si/Mo/Ti multilayer film. The thicknesses of the Mo interlayer and Ti film could be accurately measured from the cross-sectional SEM image. Table 1 tabulates the measured results of the Mo interlayer and Ti film thicknesses of the Mo-1, Mo-2, Mo-4, and Mo-8 samples. The thickness of the Mo interlayer increased from 54.3 nm to 331.5 nm with increasing Mo deposition time from 1 min to 8 min, which is in accord with the XRD results. The thicknesses of the Ti films for all four samples stayed between 650 nm to 700 nm with nearly the same deposition time. The EDS mapping results also demonstrated that a pure Ti film had been grown without the precipitation of other compounds like the Ti-Si compound [42,43]. 

**Figure 4:**
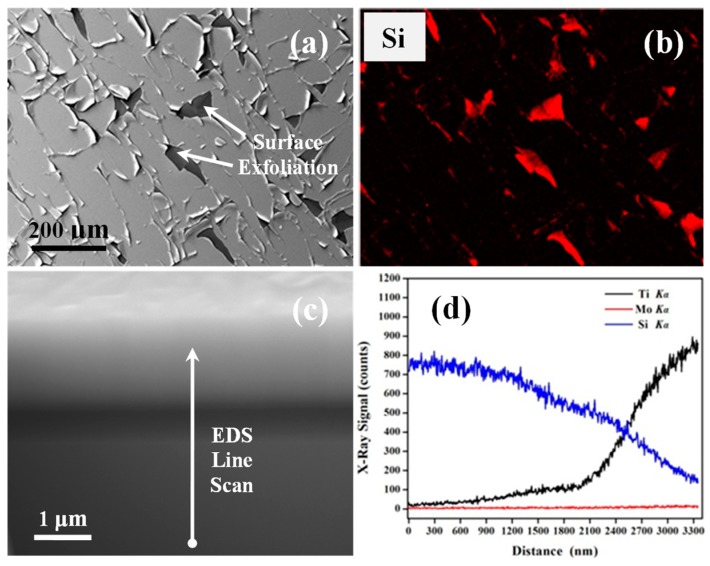
(Color online) (**a**) SEM image of Mo-8a sample taken from the delamination area and (**b**) the Si element distribution in (a). (**c**) Cross-sectional SEM image obtained from Mo layer absent area and (**d**) energy dispersive spectroscopy (EDS) line scan of Ti, Mo, and Si elements along Line 1 in (c).

To verify whether or not the surface delamination occurred during the Ti film deposition, another Mo-8a sample was fabricated with the same parameters as the Mo-8 sample, except that the thickness of the Ti film increased to around 1500 nm. Figure 4a,b show an SEM image of the Mo-8a sample which was taken from the center delamination area and the corresponding Si element EDS mapping result. It is clear that the dark areas (labeled by arrows) in Figure 4a indicate a strong Si signal, revealing that the Mo/Ti multilayer film completely peeled off from the Si substrate in those areas on the Mo-8a sample surface. Figure 4c,d exhibit a cross-sectional SEM image of the Mo-8a sample and an EDS line scan of the Ti, Mo, and Si elements along the line labeled in Figure 4c, respectively. The EDS line scan result illustrates that the Mo interlayer is absent from the multilayer film. It was demonstrated that the Ti film bonded well to the Mo film, which induced the full delamination of the Mo/Ti multilayers from the Si substrate. However, the Mo/Ti film was not firmly bonded on the Si substrate, which might have been due to the small roughness of the polished single crystal Si substrate. These results support the conclusion that the Ti film was directly deposited on the Si substrate after the Mo/Ti film started to delaminate from the Si substrate during the Ti film deposition. 

**Figure 5:**
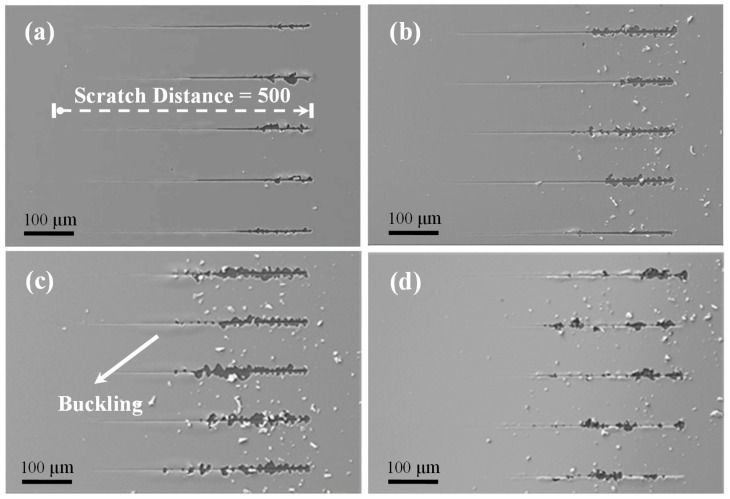
SEM images of the five scratch tracks performed on each of the (**a**) Mo-1, (**b**) Mo-2, (**c**) Mo-4, and (**d**) Mo-8 samples. The scratch tracks were explored from left to right in these SEM images to a maximal scratch distance of 500 μm.

**Figure 6:**
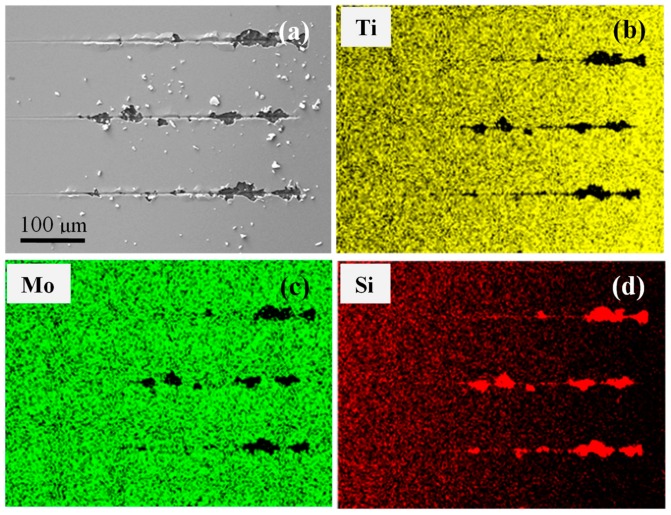
(Color online) (**a**) A magnified SEM image of three scratch tracks on the Mo-8 sample and the (**b**) Ti, (**c**) Mo, and (**d**) Si element distributions of (a).

The SEM images in Figure 5 show the five scratch tracks induced by the nano-indenter on each of the Mo-1, Mo-2, Mo-4, and Mo-8 samples. For the Mo-8 sample, the scratch experiments were explored on the area that was free of surface delamination. The scratch tests were performed from left to right with a linear increasing normal force to a maximum distance of 500 μm. For each scratch track, the width of the track steadily increased with the increasing displacement of the nano-indenter into the film surface when the film was tightly bonded to the Si substrate. However, the width and the shape of the track were sharply enlarged or the film was completely peeled off from the substrate once the film started to delaminate [49]. As shown in Figure 5a, it was clear that a slight exfoliation was induced by the nano-indenter at the end of the scratch tracks for the Mo-1 sample. With the Mo deposition time increased to 2 min, the surface exfoliation became stronger for the Mo-2 sample while a few Mo/Ti film particles could be found around the scratch tracks. It is interesting to note that the surface buckling occurred at the center of the scratch distance for the Mo-4 sample. Obviously, a severe surface exfoliation with a great number of film particles could be observed at the center of the scratch distance for the Mo-8 sample. Figure 6a displays the magnified three scratch tracks on the surface of the Mo-8 sample, and Figure 6b–d shows the EDS mapping of the Ti, Mo, and Si element distributions seen in Figure 6a. The signals of the Ti and Mo elements disappeared while the signal of the Si element changed to be become stronger in the surface exfoliation area. The EDS mapping results demonstrate that the multilayer of the Ti film and the Mo interlayer was tightly bonded and was entirely exfoliated from the Si substrate during the scratch test. 

**Figure 7:**
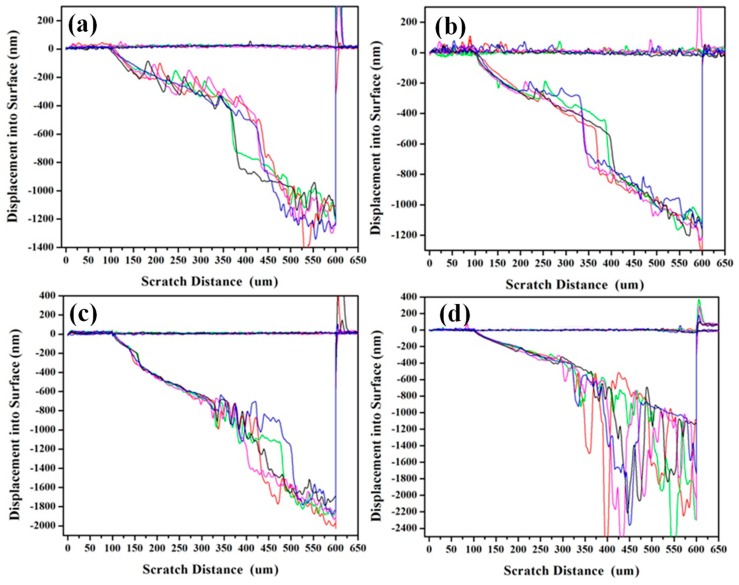
(Color online) The relationship between displacement into surface and scratch distance recorded during the scratch experiments performed for the (**a**) Mo-1, (**b**) Mo-2, (**c**) Mo-4, and (**d**) Mo-8 samples.

Figure 7 depicts the relationship curves between the displacement into the surface and scratch distance recorded during the scratch experiments performed on the Mo-1, Mo-2, Mo-4, and Mo-8 samples. All four samples had a smooth surface with slight fluctuations in displacement in the first round of the scratch scan, which were beneficial for acquiring accurate scratch results. As for the Mo-1 and Mo-2 samples, the displacement steadily increased with increasing normal force load and scratch distance where the Mo/Ti films remained attached to the Si substrate. Once the film exfoliation had taken place, the displacement sharply increased with the scratch distance. For the Mo-4 and Mo-8 samples, the displacement started to fluctuate remarkably with the scratch distance when the film started to exfoliate. The nano-indenter-induced film delamination from the scratch track was an ideal way to evaluate the film adhesion on the substrate [31,49,51]; indentation methods generally rely on the onset scratch distance to cause the film to exfoliate. The average scratch distances and displacements, where the Mo/Ti film started to delaminate from the Si substrate, were recorded for all scratch tracks and have been tabulated in Table 2. It is notable that the film exfoliation occurred at the displacement where the scratch nano-indenter was away from the interface between the Mo interlayer and Si substrate. The weak cohesion between the Mo/Ti multilayer and Si substrate might have been due to the small roughness of the Si substrate. The scratch distance decreased from 301.1 ± 29.6 nm to 224.5 ± 22.7 nm with the increasing Mo interlayer thickness, suggesting that the thicker Mo interlayer reduced the cohesion between the Mo/Ti film and the Si substrate. 

**Figure 8:**
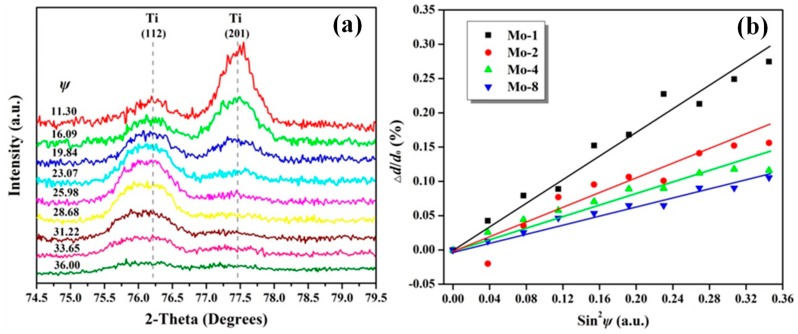
(Color online) (**a**) XRD patterns of the Mo-1 sample showing the peak shift of Ti (112) as a function of *ψ* angle. (**b**) In-plane residual stress derived from the XRD patterns in (a) using the sin^2^*ψ* method for the Mo-1, Mo-2, Mo-4, and Mo-8 samples.

Figure 8a shows the XRD patterns as a function of *ψ* angle for the representative Mo-1 sample. Two diffraction peaks of Ti (112) and Ti (201) can be found in the XRD patterns with a 2*θ* range between 74.5° and 79.5° [4]. The Ti (112) peak shifted towards a lower 2*θ* angle with increasing *ψ* angle, suggesting that the Mo-1 sample had an in-plane tensile stress. The intensity of the Ti (112) peak decreased with increasing *ψ* angle, which was ascribed to the smaller number of grains detected at higher *ψ* angles. The relationships between the 2*θ* and *ψ* angles for the Mo-1, Mo-2, Mo-4, and Mo-8 samples were acquired. To clearly distinguish the relationship between residual stress and the Mo interlayer thickness, curves of △*d*/*d* versus sin^2^*ψ* with different slopes were plotted for all four samples and are shown in Figure 8b. All date points for each of samples were linearly fitted, with the slope positively relating to the residual stress. It is apparent that the slopes of the fitted lines decreased with increasing Mo layer thickness. The residual stress values were calculated by the 2θ-sin^2^ψ method, which has been reported elsewhere [12,23]. The Young’s modulus and Poisson’s ratio parameters used were 120.2 MPa and 0.361 [52], respectively. Table 3 summarizes the calculated residual stresses of all four Si/Mo/Ti multilayer films. It is interesting to note that the residual stress decreases from 686.4 ± 40.6 MPa to 257.6 ± 14.0 MPa with an increase in Mo interlayer thickness from 54.3 nm to 331.5 nm. It is well known that the residual stress of thin film strongly correlates to the film growth mechanism [44,53], substrate temperature [26], and the discrepancy in thermal expansion coefficient between film and substrate [53]. However, the thickness of the Mo interlayer would be the critical factor affecting the residual stresses in the Si/Mo/Ti multilayer films. 

According to the XRD patterns and EDS maps shown in Figure 2 and Figure 3, the Mo films with different thicknesses were normally grown on the Si substrates despite the smooth surface of the substrates. The Ti film could also be successfully grown on the Mo film if the Mo interlayer thickness was below 139.8 nm. However, the Mo/Ti multilayers started to exfoliate from the Si substrates during the subsequent Ti film deposition, which was due to the weak cohesion between the film and substrate once the Mo interlayer thickness was thicker than 139.8 nm, as demonstrated by the EDS result of the Mo-8a sample as shown in Figure 4. It was supposed that the Mo interlayer and Ti film were both in thermal expansion states, since the substrate temperature was high, at 700 °C. The thermal expansion coefficients of Si, Mo, and Ti are 4.0 × 10^−6^/K [27], 4.8 × 10^−6^/K, and 8.6 × 10^−6^/K [54], respectively. The Ti film delaminated from the substrate during the Ti film deposition, which was most probably due to the higher thermal expansion coefficient of Ti compared to those of Mo and Si. The thermal expansion of the Ti film was constrained by the Mo interlayer caused the Ti film delamination [28,53]. The following Ti film was grown directly on the Si substrate to form the Ti-Si complex [42,43], which alternately reduced the quality of the Ti film. The growth of high-quality Mo film on Si was attributed to their similar thermal expansion coefficients. 

Dauskardt et al. [55] has suggested that the debonding of multilayer thin film structures is driven by residual stresses, including the intrinsic growth stresses produced during deposition and the thermal expansion mismatch stresses. On the other hand, the thickness of the Mo interlayer was also one important factor affecting the cohesion between the Mo/Ti film and Si substrate, with the thicker Mo layer reducing their cohesion. Chason et al. [44] have reported that compressive stress keeps increasing with increasing film thickness, while Xi et al. [56] have found that residual stress transforms from compressive to tensile and that tensile stress increases with increasing film thickness. Leterrier et al. [29] have verified that the cohesive strength and crack onset strains of SiO*_x_* film on polymer substrate decrease with increasing coating thickness. Roshangias et al. [31] have disclosed that the adhesion of TiW coatings on Si substrates decrease significantly with the increasing thickness of coatings. It can be concluded from these results that the compressive or tensile stress increases with film thickness, originating from the Ti film growth and the discrepancy of the thermal expansion coefficients of the Si, Mo, and Ti materials, which reduces the adhesive strength between the Ti film and Si substrate.

After deposition, the as-grown Si/Mo/Ti multilayer films were naturally cooled in a high vacuum environment of 2 × 10^−4^ Pa. The Ti and Mo films started to shrink during cooling. Similarly, the shrinkage of the Ti film must have been larger than that of the Mo film due to the higher thermal expansion coefficient of Ti metal. The shrinkage of the Ti film induced the generation of tensile residual stress [6,44], which was proven by XRD stress analysis. It has been demonstrated by Dehm et al. [28] that the difference in thermal expansion coefficients can induce thermal compressive stress in films if the thermal expansion coefficient of film material is higher than that of the substrate. However, the residual stress becomes tensile with the occurrence of isolated cluster coalescence [6,44] and the subsequent increasing of film thickness [28]. Chason et al. [44] and Floro et al. [45] have demonstrated that residual stress in films undergoes typical compressive and tensile steps. Film stress is compressive during the early stage of nucleation and later becomes tensile, which is associated with the formation of grain boundaries. The Mo/Ti multilayer film delaminates from the Si substrate if the film is not tightly bonded to the substrate. The XRD stress results demonstrated that the Si/Mo/Ti samples with thicker Mo interlayers have lower residual tensile stress. This could be explained by the fact that the released residual stress provided the driving force for the film exfoliation and the film exfoliation relaxed the residual tensile stress of the Ti film [55]. The above results suggest that the thermal expansion coefficient of the film and the substrate materials and the design of the film thickness are important factors affecting the film quality in the design of multilayer films. 

## 4. Conclusions

In conclusion, pure Ti films were successfully grown on single crystal Si substrates by the introduction of Mo interlayers. The design of an Si/Mo/Ti multilayer film suppressed the chemical reaction between the Ti film and Si substrate. It was found that the thickness of the Mo interlayer played an important role in the cohesion between the Mo/Ti multilayer film and the Si substrate, which significantly decreased with increasing Mo interlayer thickness. All the Si/Mo/Ti multilayer films presented in-plane tensile residual stresses which might be due to the lattice expansion at a high film growth temperature of 700 °C and the discrepancy in thermal expansion coefficients between the Ti film and Si substrate. The tensile stress, derived from the XRD stress analysis results, decreased from 686.4 ± 40.6 MPa to 257.6 ± 14.0 MPa when the Mo interlayer thickness increased from 54.3 nm to 331.5 nm. The decreased tensile stress during the Si substrate cooling process provided the driving force to reduce the cohesion between the Mo/Ti film and Si substrate for the thicker Mo interlayer samples. 

## Figures and Tables

**Table 1:** Sample designations of Si/Mo/Ti multilayer films with different Mo deposition times and thicknesses of Mo and Ti films measured by scanning electron microscopy (SEM).

Sample Designation	Mo-1	Mo-2	Mo-4	Mo-8
Deposition time of Mo films (min)	1	2	4	8
Thickness of Mo films (nm)	54.3	103.7	139.8	331.5
Thickness of Ti films (nm)	692.0	657.0	654.3	693.2

**Table 2:** The average scratch distance and displacement into the surface of five scratch tracks on the Si/Mo/Ti multilayer film surface where the Mo/Ti film started to delaminate from the Si substrate. The standard deviation was statistically calculated from the five scratch tracks for each sample.

Sample Number	Mo-1	Mo-2	Mo-4	Mo-8
Scratch distance (nm)	301.1 ± 29.6	262.1 ± 26.7	227.4 ± 8.7	224.5 ± 22.7
Displacement into surface (nm)	469.4 ± 51.0	433.8 ± 92.1	689.3 ± 27.6	419.6 ± 63.0

**Table 3:** The residual stresses of the Si/Mo/Ti multilayer films determined by XRD using the sin^2^*ψ* method. The standard deviation was obtained by fitting the data points of *△d*/*d* verse sin^2^*ψ* using the linear least square method.

Sample Number	Mo-1	Mo-2	Mo-4	Mo-8
Residual stress (MPa)	686.4 ± 40.6	395.1 ± 34.6	294.0 ± 22.2	257.6 ± 14.0
